# Timely Euthanasia in the United States Dairy Industry–Challenges and a Path Forward

**DOI:** 10.3390/ani10010071

**Published:** 2019-12-31

**Authors:** Jennifer B. Walker, I. Noa Roman-Muniz, Lily N Edwards-Callaway

**Affiliations:** 1 Danone North America, 3333 Dan Morton Drive, Dallas, TX 75236, USA; Jennifer.Walker@danone.com; 2 Department of Animal Science, Colorado State University, Fort Collins, CO 80523-1171, USA; Noa.Roman-Muniz@colostate.edu

**Keywords:** culling, dairy cattle, human-animal bond, timely euthanasia, welfare

## Abstract

**Simple Summary:**

Euthanasia is a critical component of livestock production systems, providing producers, veterinarians, and caretakers a way to alleviate animal suffering. Although the intention is to keep all animals healthy, there are times when an animal must be euthanized when chances of recovery are low, the animal’s pain is not manageable, and/or its quality of life has deteriorated. The timeliness of euthanasia is an equally critical component of animal welfare. Activist groups have called out the dairy industry on this issue and the data suggests the untimeliness of euthanasia on dairy farms is an area in need of significant improvement. Failure to make timely euthanasia decisions impacts cow welfare and has a negative effect on the dairy business and caretaker. There are many factors influencing timely euthanasia decisions, which may be why effectively managing euthanasia on dairies can be a challenge. Factors impacting euthanasia decisions include caretaker training, availability of protocols, treatment decisions, quality of life assessment, human–animal bond, and economic influences. The dairy industry must develop effective resources to ensure a culture of care that supports timely euthanasia as a priority. This review discusses some of the factors impacting timely euthanasia decisions and proposes a path forward to improvement.

**Abstract:**

Euthanasia is a valuable management tool utilized on dairies to end the suffering of sick or debilitated cows. Euthanasia should be implemented if an animal’s pain cannot be adequately alleviated and if there is a limited chance of recovery. To be humane, euthanasia should be quick, painless, and administered by a trained individual. Despite its importance in ensuring cow well-being, the timeliness with which euthanasia decisions are made for dairy cattle is often overlooked or neglected. The timeliness of euthanasia is as important as the efficient, rapid administration of euthanasia itself. Timely euthanasia is a critical component of many on-farm animal care and verification programs yet opportunities exist within the industry to improve how effectively the industry is executing this critical component of cow management. There are challenges associated with performing euthanasia in a timely manner, such as inconsistencies in treatment protocols, inadequate employee training, difficulties assessing a cow’s quality of life, and impacts of the human–animal bond on decision-making. The objective of this paper is to explain the importance of timely euthanasia to dairy cattle welfare, identify the challenges that can prevent the timeliness of euthanasia, and provide solutions and practical suggestions for improving the management of timely euthanasia on dairies.

## 1. Introduction

Euthanasia marks the humane endpoint of an animal’s life, a “good death” bestowed upon an animal when pain and suffering cannot adequately be alleviated and if there is little chance of recovery. Euthanasia is a tool that veterinarians and producers can use to end animal discomfort and pain from an unrecoverable disease and poor quality of life. All animal industries utilize euthanasia: in research, wildlife management, companion animal medicine, and food animal production, to name a few. Interestingly, people often employ euphemisms to replace the term euthanasia, such as “putting an animal to sleep”, “sacrificing an animal”, or “putting an animal down”, to potentially lessen the emotive response associated with the necessary but sometimes burdensome undertaking of performing euthanasia [1]. Despite differences between industries, many of the challenges surrounding euthanasia are the same, including but not limited to appropriate training, caretaker compassion fatigue, timeliness of the decision to euthanize based on owner demands and values, financial reasons, and consistency in protocols for determining clinical endpoints. Despite knowing that euthanasia may be the most humane choice for an animal, electing and performing euthanasia can cause animal caretakers and owners to experience “moral stress” [2], particularly for those who work daily to keep animals under their care alive and well. Caretakers tend to animals daily and sometimes they may view euthanasia as a failure in their ability to provide adequate care, making it emotionally tasking, in both arriving at the decision and performing the procedure [3]. Often caretakers may avoid euthanasia of animals in need because they sincerely are hoping the animal may heal and recover, not because they want the animal to suffer.

In the dairy industry, timely euthanasia is viewed as a critical component of dairy cattle management yet, based on the available data [4], untimeliness of euthanasia on dairy farms has been identified as an area in need of significant improvement. Identifying the right time to euthanize a dairy cow can be a challenge as owners, veterinarians, and caretakers can have different ideas of how to determine appropriate humane endpoints. Humane endpoints can be influenced by many factors, whether it be the relationship with the cow, the understanding of disease progression and prognosis, or the financial burden associated with animal production, to name a few. Timely euthanasia, in dairy cows, heifers, and calves alike, is a complex, impassioned topic that is impacted by many factors, some of which will be discussed in detail in this review. Having the ability to perform euthanasia to end animal suffering is a critical component of animal management systems, but it has to be implemented appropriately and in a timely manner with the consideration of the cow’s welfare in the forefront of decision-making.

## 2. What is “Timely Euthanasia”

The meaning of euthanasia is widely understood; every paper on the topic, no matter the animal industry, provides a definition of the word itself. What many seem to struggle with is identifying the right time to euthanize a compromised and suffering animal, in companion and food animal industries alike. Despite the recognized importance of timely euthanasia and the frequent use of the word “timely” within the dairy industry, it could be argued that there is not a shared understanding of what “timely” actually means when it comes to making euthanasia decisions. One could presume that there is a common understanding of the word “timely” based on how it is used in the English language; timely means promptly, occurring sufficiently early, opportunely, and appropriately, all equally as vague and open to interpretation as “timely”. As a result, in practice, unfortunately, these definitions do not always translate into the same interpretation, procedure, and outcome for the cows in question.

In the United States, standards for animal care created and implemented by industry associations provide provisions for euthanasia across food animal systems, including requirements for training and protocols specific to euthanasia [5,6,7]. Focusing on dairy cattle care programs, the FARM program (Farmers Assuring Responsible Management), overseen by National Milk Producers’ Federation (NMPF), indicates within their education handbook (FARM Version 3.0; Version 4.0 will be released January 2020) that euthanasia is appropriate “when an animal’s quality of life is decreased or when pain and suffering cannot be alleviated” [5]. Some examples of situations when euthanasia would be a prudent choice are also provided within the manual, including when an animal appears to be experiencing severe pain or distress, cannot be saved or moved properly, has been chronically ill, or was recently treated with antibiotics requiring an extended withholding period. The FARM manual references other established industry recommendations, specifically the American Veterinary Medical Association (AVMA) and the American Association of Bovine Practitioners (AABP), for guidance on decision-making and procedures for implementing euthanasia [8,9]. Although the FARM manual includes indicators and guidance materials for euthanasia decision-making, it lacks strong messaging about the timeliness of decision-making and how critical that is for preventing animal suffering. For example, the manual states euthanasia should be administered “in a timely manner, if warranted” but does not include any reference to what that actually means in practice. Likewise, the AABP guidance document on humane euthanasia of cattle fails to mention the timeliness of euthanasia decision-making at all [8]. Failing to provide “humane euthanasia” is considered “willful mistreatment” of animals within the FARM program, as well as the BQA (Beef Quality Assurance) program, but there is little description of what constitutes inhumane euthanasia, i.e., is untimeliness considered inhumane? [5,6]. The Common Swine Industry Audit and the Pork Quality Assurance Plus program managed by the National Pork Board include more specific provisions for the “timeliness” of euthanasia, including explicit time frames for euthanasia for certain animal conditions and the inclusion of a lack of timely euthanasia as a critical audit failure, e.g., “animals that have no prospect for improvement or not responding to care and treatment after two days of intensive care must be humanely euthanized” [7,10]. Additionally, the DairyWell audit tool, developed and applied by Dean Foods (Dallas, TX, USA) on the independent contract dairy farms that supply to their processing facilities, provides a definition of timely euthanasia for moribund and injured dairy cattle, including language with more urgency although still open to interpretation, e.g., animals needing euthanasia “must receive immediate action which includes either prompt medical treatment by a veterinarian or euthanasia” [11]. Even with specific language included in training materials, dairy workers have anecdotally shared with authors that depending on other farm priorities, “immediate” could mean “after I finish this other task”. In conversations with some dairy employees, there is an idea that euthanasia takes place according to a schedule (e.g., “we euthanize cows at 2 p.m. when that person shows up for work”) instead of according to need. Perhaps “timeliness” is a component of euthanasia that is taken for granted, sometimes overlooked relative to other competing priorities, and needs to be more clearly articulated in educational materials and training provided to dairy employees.

Generally, the food animal industries recognize that timely euthanasia is critical to maintaining animal welfare, yet both lack a clear understanding of what timely means and a clear message of how important being timely is in managing dairy cow welfare. Jasani [12] delivered a talk at the British Small Animal Veterinary Association Congress entitled “Better a week early than a day too late: euthanasia”, an extremely poignant phrase in the context of this topic. Euthanasia is a critical decision in regard to an animal’s welfare, a decision better made early than too late. Jasani [12] provided discussion points and questions focused on understanding the right time for euthanasia, understanding suffering, and identifying how much suffering is too much. When identifying the “right” time for euthanasia, are we (the dairy industry) defining the “right” time as what is better for the dairy cow or what is better for the caretaker, the practice, or the other priorities on the farm? When euthanasia is not timely, the suffering is that of the dairy cow, regardless of what challenges are associated with making the decision, and that must be remembered. It is important to note that the “untimeliness” of euthanasia decisions on dairy farms is rarely related to a lack of compassion of individuals but associated with other challenges, such as training, culture, knowledge, and bonds with the cows, all to be discussed in further sections.

## 3. How Timely Euthanasia and Culling Decisions Are Intertwined

Culling decisions and euthanasia decisions are interconnected, the shared theme being that the industry must make better and more prompt decisions in regard to both. In the United States, nearly one third of dairy cows are culled from farms annually and the majority of these cows are sent to slaughter either via an auction market or directly to a slaughter facility [13]. Many culled cows are in satisfactory condition and are a result of voluntary culling, a decision based on productivity, not on an underlying health concern and certainly not animals that were candidates for euthanasia. There is another group of cows in this culled population that are an outcome of involuntary culling decisions, those that are culled as a result of a health issue, such as infertility, lameness, mastitis, or injury. Often these cows are in a compromised condition and may deteriorate quickly. Several benchmarking studies, described in detail below, have characterized the condition of cull cows arriving at slaughter plants, noting conditions with grave welfare implications for the cow [4,14,15]. We argue that some of these culling decisions should actually be euthanasia decisions. Although the majority of culled cattle arriving at slaughter facilities are in adequate condition and are fit for transport [14], not all culled cattle should be sold into the supply chain. If they are suffering and not fit for transport, producers should elect to euthanize these animals, rather than sell them. Additionally, the euthanasia decision should be made even sooner and not be just a consequence of a culling decision, as this could be too late in regards to the animal’s welfare. Sending a cull cow to slaughter is a suitable option only if the cow is not suffering and is fit to be transported. “Proactive culling” is a practice introduced here by the authors as a deliberate, timely practice of identifying cattle for sale based on their state of welfare, likelihood of recovery, and quality as a beef animal rather than their state of production or market value.

## 4. Animal-Focused Data and the Impacts of Untimely Euthanasia

To understand the magnitude of dairy cattle impacted by untimely euthanasia decisions, the condition of cattle sent to slaughter and the number of dairy cattle that die on the farm must be examined, across every age group, including pre-weaned calves, although the authors are not aware of available data reporting the condition of calves at slaughter. A survey conducted to benchmark animal welfare at slaughter plants supplying a specific multi-national company provides data on the condition of mature dairy cattle upon arrival to slaughter [15]. This survey focused on severe conditions, that is, cattle that were considered unfit for transport and should have either been marketed sooner or euthanized on-farm. Outcomes measured included: severe lameness, body condition score, udder condition, uterine prolapse, cancer eye, malaise, wounds, active parturition, nervous system disorder, and non-ambulatory, many of which are included as parameters to assess fitness for transport in industry guidelines [5,8]. The survey reported that within the United States, 9% of the dairy cattle observed had one or more severe welfare conditions. Although 9% may seem to be a low number empirically, considering over 3 million cull dairy cows are marketed each year [16], 9% translates into approximately 300,000 dairy cows each year that endure the journey from farm to market to slaughter while suffering from a painful condition. The journey to slaughter for culled dairy cows is difficult to quantify as the majority of cull cows are transported to an auction market prior to being delivered to a slaughter facility [4]. The 2016 National Beef Quality Audit, a national benchmarking study, indicated that in the study population, the average transport time from the last stop prior to the slaughter plant was 6.7 h [14], but this likely does not represent the total transportation duration. Simply put, these compromised cows represent close to one-half-million bad decisions on the part of dairy farmers, veterinarians, and cattle buyers.

The USDA National Animal Health Monitoring System (NAHMS), which collects farm-reported estimates of animal health data, is consistent with the report by Vogel et al. [15]. Estimates of the number of dairy cattle affected by severe conditions with subsequent recovery, sale, and mortality rates is summarized in Table 1 using data from the 2014 NAHMS Dairy Report [4]. Data indicate that 10% of the cattle removed from dairy farms were removed because of the following severe conditions: cancer eye, bloat, bloody gut, or being considered non-ambulatory. Lame cows accounted for 16.8% of cows removed from dairy farms. Severe lameness was not distinguished from moderate lameness in the NAHMS study [4]. The distinction between moderate and severe lameness is important; moderately lame cows are considered fit for transport while severely lame cows are not. While acute moderate lameness is reasonably responsive to treatment, severe lameness, especially that resulting from failure of prompt medical attention and involving tissues deep in the foot, is often refractory, becoming chronic and causing severe weight loss as a result of a cow’s inability to compete at the feed bunk and unwillingness to stand, electing for the opportunity for rest over the opportunity to eat. Applying published studies surveying US cattle it is estimated that 7% of the reported 16.8% of cows affected by lameness are likely to be considered severely lame [17]. Using NAHMS data applied to the 9,399,000 head of dairy cattle in the US [18], it is estimated that over 300,000 cattle unfit for transport were sent to market. The NAHMS data did not include identical condition classifications as Vogel et al. [15], which may explain the difference in estimates of the total number of un-fit cows sent to market.

Defining the number of cattle not-fit for transport sent to slaughter is critical to developing better decision-making guidelines about timely euthanasia. Equally important is defining the number of cattle that die on-farm without the benefit of euthanasia, as allowing an animal to suffer through the process of dying is the antithesis of timely. The NAHMS data [4] summarized in Table 2 reports the number of cattle that die on farm for each age group, lactating cows, heifers, and preweaned heifers. The NAHMS data demonstrate poor practices specific to timely euthanasia in lactating dairy cattle in addition to highlighting the sensitive issue of poor euthanasia practices in pre-weaned and heifer groups. The NAHMS data [4] suggest that less than 1.0% of the nearly one-half million calves and heifers that die on dairies annually are provided the benefit of euthanasia. This figure illustrates a critical failure on the part of farm management to recognize the suffering that often accompanies a natural death. Pre-weaned calves represented a particularly sensitive failure, with only 0.4% of the 6.4% reported mortalities having been euthanized (Table 2). Given that the majority of calf mortality is the result of chronic illness (diarrhea or pneumonia), where there is ample opportunity for caregivers to evaluate the condition, appetite, energy, and hydration of calves twice daily, and take action to ensure calves are not allowed to expire on their own, it seems inexcusable that 95% of calves that die on farm each year suffer the process of death without the benefit of euthanasia.

## 5. Industry-Focused Impacts of Untimely Euthanasia

The direct negative impacts of untimely euthanasia to the cow or calf are clear. It is neither possible nor productive to compare the suffering of an unassisted death to the suffering associated with a cow unfit for transport enduring the journey from farm to market to slaughter, both are unacceptable in regard to dairy cattle welfare. It is, however, possible to define the suffering in both instances as unnecessary and avoidable. That definition alone should be enough to compel the people within the dairy industry to reflect and change behavior. The impact of these critical failures in administering timely euthanasia on the dairy industry is also worth discussion. Recognizing how performance in this area impacts the industry, farm, and the employee should sound an alarm and are key to understanding how and if there is to be meaningful change at the farm level.

### 5.1. The Risk to the Business

While the impact of poor performance on the dairy industry specific to timely euthanasia is difficult to quantify, it is important for dairy farmers to appreciate that supplier performance in this specific area can negatively impact customers, that is, either the immediate processor of raw milk or the secondary customer responsible for selling milk to consumers through various supply chains. The use of undercover videos has become a method utilized by animal rights activist organizations to expose poor practices on farms and influence the supply chain to make policies regarding animal welfare. Specific to the dairy industry, the impact of a supplier failure, in this case the dairy farm, on its customer(s) has been amplified following the dissemination by animal rights activist organizations of four separate undercover video investigations in 2017 and again in 2019. Videos released presented evidence of veal calves, calves raised at a calf ranch, or calves raised on a dairy in addition to adult cattle on dairy farms that were not provided timely euthanasia. In response to the video release, retailers suspended milk pickups from the farms in question and some pulled associated brand product off the shelves [19,20]. The videos also caused some customers to revise their animal welfare audit programs and policy [21,22], making programs more robust by adding additional oversight, such as third-party verification and video monitoring, all resulting in increased business expenditures. It has been estimated that an individual farm audit costs as much as $1500 [23]. Depending on the processor and the number of farms in their supply, implementing annual third-party audits can therefore represent a significant drain on budgets in addition to limiting the flexibility of procurement in accommodating swings in supply and demand, resulting in additional supply management overhead. In addition to the financial loss related to decreased sales, product recall/destruction, and cancelled customer contracts, which are difficult to quantify and not shared publicly, an additional risk in the form of consumer litigation alleging consumer fraud has emerged as a new and potentially significant financial risk [24,25]. Legal jeopardy is not limited to the supply chain business unit. While it has not been the norm, farm employees captured in the videos are at risk of prosecution. In 2017, a total of five arrests were made in relation to one undercover video [26], and the most recent 2019 video resulted in a single suspect being arrested and charged with felony animal torture and a misdemeanor for animal cruelty while warrants for two additional employees were outstanding [27].

### 5.2. The Risk to the Caretakers

Beyond fear of prosecution, there are other unrecognized or at least poorly acknowledged risks to the farm employee when euthanasia is not timely. Animal caretakers are directly impacted by euthanasia decisions. The “moral stress” [2] of performing euthanasia can cause animal caretakers to experience psychological, emotional, and physical ailments [28,29]. Considerable research has been conducted exploring the impacts of performing euthanasia on people who have chosen careers based on their affinity for caring for animals, such as veterinarians, animal shelter workers, livestock owners, and farm employees [30,31]. Reeve et al. [30] indicated that employees involved in euthanasia showed significantly greater levels of work stress, stress-induced physical ailments, work-to-family conflict, and dissatisfaction with their work. In a review of the effects of euthanasia on occupational stress at shelters, clinics, and biomedical research centers, Scotney et al. [32] identified that employees directly involved in euthanasia report significantly greater levels of work stress and lower job satisfaction, which may result in greater employee turnover and psychological distress. McDiarmid [33] reported that employees at animal shelters who perform euthanasia are at risk for high blood pressure, ulcers, unresolved grief, depression, drug abuse, and suicide. In another study, 74% of shelter managers agreed that performing euthanasia contributes to employee burnout, and 26% of shelter managers believed that performing euthanasia contributes to employee turnover [34]. Additionally, animal caretakers have reported impacts on their personal lives and feelings and expression of sadness, anger, and depression as a result of euthanizing the animals for which they provide care [34,35].

Individuals cope with work-related stress in different ways [36], and there are many different factors influencing both coping strategy and coping strategy success [31]. Focus groups facilitated on dairy operations in the Western US [37] revealed the perceptions of caretakers regarding cow and calf euthanasia. While workers understand that timely euthanasia is a kind way to cease animal suffering and the right thing to do for a sick animal in pain and a poor prognosis, they admit that making that decision is extremely hard, especially when dealing with younger animals or animals that they feel a connection to. Although participants mentioned several ways in which they deal with the stress of tending to sick animals and having to perform euthanasia, professional services to help caretakers are not a resource that is well known in the rural areas where the focus groups were conducted. It is evident that there are impacts of performing euthanasia on worker well-being and this can have a greater impact on the sustainability of the dairy operation itself if not addressed; it is necessary to foster a culture of caring for both the caretakers and the animals. The impacts of euthanasia on the animals, the workers, and the dairy business are substantial and can have significant impacts on all parties.

## 6. What Are the Factors Impacting Euthanasia Decisions?

There are many factors that influence timely euthanasia decisions on farms, which could in part be why effectively managing euthanasia on dairies is sometimes a challenge. Focus groups conducted with swine industry experts identified logistical challenges, balancing competing priorities, economic considerations, emotional strain on caretakers and farm culture, and accountability as barriers to timely euthanasia on swine farms [3]. Although research exists that explores the challenges and factors associated with the management of euthanasia in companion animal and shelter medicine and biomedical science [2,32,38,39], little has been investigated regarding the barriers to euthanasia in food animals [3,35], particularly in the dairy cattle industry. Several significant barriers to timely euthanasia on dairy farms will be discussed below.

### 6.1. Inconsistent Caretaker Training

While the dairy industry continues to consolidate and larger herds become more reliant on hired labor to provide animal care, research continues to indicate that development and dissemination of effective training programs is an area of critical need for dairy operations. One study indicated that dairy workers report a lack of availability and consistency of training and have cited understaffing, time pressures, and poor supervisor communication skills as causes for training deficits [40]. Most dairy workers responsible for animal care are foreign-born Latinx individuals with diverse backgrounds, and varying levels of formal education, livestock experience, English language skills, and acculturation [40,41]. This diverse population of caretakers has shown serious knowledge gaps in the areas of animal behavior and physiological processes [40], and require training on basic and complex topics to be better able to make decisions that affect animal wellbeing, including whether to continue medical treatment or to consider euthanasia.

Although various training programs are available online and in other formats, and have shown effectiveness in improving animal welfare-related knowledge [42], the aforementioned NAHMS survey revealed that only 33.4% of dairy operations train their caretakers in the management of non-ambulatory cows and 20% provide training in euthanasia [4]. Hoe and Ruegg [43] reported that in 13 out of every 100 euthanasias, dairy personnel without training performed the task and that veterinarians were involved in euthanasia decision-making less than one third of the time. As highlighted earlier, only 40.6% of all the dairy cows that died on the farm in 2013 were euthanized [4]. These findings underscore the need for training programs that teach dairy workers how to properly euthanize cows, and more importantly, offer guidelines to aid with decision making to decrease the number of cows that die on the farm without timely humane assistance. These training programs should be developed and facilitated with the input of veterinarians, dairy producers, and workers to maximize engagement and enhance cultural congruency (alignment with the values, needs, and perspectives of learners) and effectiveness.

### 6.2. Lack of Written Standard Operating Procedures

In addition to the lack of availability and consistency of training sessions that effectively address decision-making and euthanasia procedures, an impediment to timely euthanasia of dairy cows is the lack of written and clear standard operating procedures (SOPs) on farms. Written guidelines are lacking on a majority of dairy farms for even the most critical of medical conditions managed on the majority of farms, such as non-ambulatory animals [44]. Although written protocols are essential for many on-farm procedures, protocols are particularly critical for procedures like euthanasia because the repercussions if performed incorrectly (e.g., wrong method, incorrect placement, untimeliness) are grave for the animal and potentially the worker as it relates to human safety. Managing non-ambulatory animals is challenging on any farm and warrants discussion of continued treatment versus euthanasia, and yet, while 76.5% of dairy operations surveyed in 2014 had at least one non-ambulatory cow, 22.6% of them had written guidelines for their care [43]. The same operations reported that less than half of non-ambulatory cows that died on the farm were euthanized and that only 59.1% of those euthanized were non-ambulatory for two days prior to euthanasia being performed. The reality is, based on experience and published data, that except for hypocalcemia, the chances of a non-ambulatory cow recovering are slim, ranging from 9.3% to 18.4% [45], and that cows non-ambulatory >24 h had a recovery rate of 8.2%. Considering this evidence, it would seem prudent for a farm adopt strict time limits for how long a cow is allowed to remain non-ambulatory.

Beyond protocols for managing compromised cattle, including clear time-bound decision guidelines for euthanasia, farms often lack farm specific guidelines for managing euthanasia in general. Although some groups offer algorithms for the decision-making process and cite unfitness for transport, likely condemnation at slaughter, or conditions that are untreatable or unresponsive as indications for immediate euthanasia [46], very few farms offer this level of direction to caretakers. This lack of specific guidance introduces ambiguity for caretakers who face competing priorities, and pressure and unclear direction from management as they try to provide timely euthanasia [37].

### 6.3. To Treat or Not to Treat

Removing the ambiguity experienced by animal caretakers by providing written SOPs and training is only part of the puzzle. Direction given to animal caretakers must be developed with the end in mind taking into account the likelihood of treatment success and return to production, not simply the likelihood of salvage or survival. As introduced in a previous section, of the conditions determining a cow’s fitness for transport or need for euthanasia (e.g., severe lameness due to deep sepsis of the digit, advanced cancer eye, non-ambulatory, bloody gut, and bloat [4,47,48,49]), most are considered refractory to treatment unless treated at the earliest signs of disease. In these instances, we argue that in the majority of cases, timely euthanasia is the right decision from an ethical and business perspective. The business case was presented earlier, highlighting the risk to the farm and the customer. The ethical case requires additional consideration. Beyond the likelihood of recovery, when a farmer or veterinarian elects to treat a cow, whether or not the pain associated with the condition can be managed must be considered.

The opportunity to provide effective pain relief to cattle is limited by both product efficacy and availability. Currently there is only one approved analgesic drug specifically approved by the Food and Drug Administration (FDA) for the treatment of pain in livestock. To be clear, lack of FDA approval does not absolutely preclude the use of a drug, it simply means that to use a drug in what would be considered an extra-label manner, the prescribing veterinarian must follow the rules set forth in the Animal Medicinal Drug Use Clarification act of 1994, which include ensuring that treatment does not result in milk or meat residues [50]. Drugs at the disposal of the licensed veterinarian to manage pain in cattle include non-steroidal anti-inflammatories (NSAIDs), opioids (example butorphanol), alpha-2 agonists (example, xylazine), and *N*-methyl d-aspartate receptor antagonists (NMDA agonists, for example, ketamine). Opioids and NMDA agonists are both controlled substances requiring a specific license to use and must be used under the control of the veterinarian. While effective for the management of procedural and post-procedural pain associated with surgical and other painful procedures, the effectiveness of these drugs in managing chronic pain is questionable [51]. The pain associated with the severe conditions leading to euthanasia is often the result of tissue or nerve damage, persistent and associated with pain hypersensitivity. While the inflammatory pain associated with mild lameness may respond modestly to NSAIDs that last from 6 to 24 h [52], severe conditions result in pain due to nerve damage that is refractory to treatment with NSAIDs and opioids [51]. To be effective, treatment would require frequent dosing (every 8 to 12 h), chronic exposure to which may result in other complications, including gastric ulcers and decreased gastric motility.

In addition to efficacy and the need for frequent dosing, the cost of treatment is certainly a consideration but second to our ability to effectively manage pain throughout recovery, making sure there is a clear definition of what success looks like. If success is a return to production following a well-managed treatment and recovery, one could argue that the decision to treat can be made on a farm-by-farm basis taking into consideration the value of the cow, the cow–caretaker relationship, and farm resources. If success is equated to whether or not a cow will be able to walk onto a trailer to be sold, at best realizing some monetary return or at worst merely avoiding the cost of disposal, we argue that the decision to treat should be abandoned in favor of timely euthanasia and investing efforts in prevention, addressing the condition sooner, or proactive culling.

It is also important to recognize that choosing to begin treatment does not signal an end to the daily evaluation and consideration of timely euthanasia as the acceptable end point. There are times when cows, treated with the best of intentions, do not recover as hoped or anticipated. In these instances, it is not uncommon to see futile treatment efforts continue, additional treatments initiated, and, in the end, the cow is kept on farm until the drug withdrawal period has been met at which time they are sent to market or slaughter even if not fit for transport. Without question, withdrawal times should be considered when making proactive culling decisions. However, dairy cattle that have not responded to treatment as expected should not be made to endure additional suffering while waiting out the meat withholding requirements to capture the salvage value of the cow or avoid other financial losses. The challenge of recognizing the conflict that exists on the farm, between making the moral judgment to provide timely euthanasia and accepting the financial loss to the business of the farming operation, deserves further consideration and is an area for future exploration.

### 6.4. Economic Influences

To develop better decision-making habits on the farm specific to timely euthanasia, it is necessary to identify the current environment that may be incentivizing the bad decisions. Compromised dairy cattle, unfit for transport, that should have been euthanized on the farm are offered for sale because there is no significant disincentive for selling and/or purchasing them. Despite their condition, there is a financial gain (or at least not a financial loss) made by not euthanizing these cows and sending them to market. In short, the current cull cow marketing system does not in practice discourage the shipment of dairy cows that are unfit for transport. As mentioned, there is significant discussion about fitness for transport and timely euthanasia within the dairy industry yet there still exists enough financial benefit within the system that results in decision-making that is not central to minimizing cow suffering.

Edwards-Callaway et al. [53] provide a summary of the market dis/incentives related to cull cow transport, sale, and purchasing decisions. Although the decision to not euthanize a cow and send her to a sale barn or slaughter plant begins on the dairy farm, it is a burden shared by the entire supply chain (e.g., livestock market owners, cattle buyers, transporters, packing facilities). Although there are risks associated with transporting and purchasing compromised cows, such as death during the process resulting in a complete loss or potential regulatory action from the handling of compromised animals, there are still benefits that often outweigh the risks, including but not limited to cost savings associated with carcass disposal, sale price of a cow even if minimal, maintaining business relationships by accepting cows of questionable condition, and the potential profit margin associated with processing lean cows [53].

### 6.5. Quality Over Quantity

Humans as a whole are not comfortable with death, often resisting it for as long as possible. Not acceptable in humans in most countries, euthanasia is generally accepted in non-human animals. This dichotomy presents an interesting question. How do we know when it is time to euthanize an animal? In companion animal medicine, when that question is asked, the common answer is that it is time when an animal no longer has a good quality of life. Quality of life (QoL) assessment is multi-dimensional, relies on the subjective experience of an individual, and thus can be a challenge to assess, even in humans. The World Health Organization (WHO) defines QoL as “an individual’s perception of their position in life in the context of the culture and value systems in which they live and in relation to their goals, expectations, standards and concerns. It is a broad ranging concept affected in a complex way by the person’s physical health, psychological state, personal beliefs, social relationships and their relationship to salient features of their environment” [54]. In human medicine, QoL assessment is used to evaluate the effectiveness of therapeutic interventions and predict patient outcomes. Although QoL assessments are used extensively in human medicine, there is no consensus on what the definition of QoL is nor is there agreement on the best method of assessment to utilize, meaning often multiple types of assessments are utilized (e.g., questions, scales with multiple categories, focus of attention) [55]. In a review published several years ago compiling all publications related to QoL assessment in humans, Gill and Feinstein [55] identified the use of 159 different QoL assessment tools and included articles that averaged 3 different tools within a study, with a range of use between 1 and 19. It appears that within human medicine, although there are numerous validated tools available, many are not explained well, only focus on medical outcomes rather than having a holistic approach to QoL, and can be labor intensive [55].

Applying the QoL definition to animals poses a bit of a challenge as our abilities to understand how an animal perceives its own life is rather intangible and relies on outward signs or observations. Although more limited as compared to human medicine, there do exist multiple approaches to assessing QoL in veterinary medicine as well. Due to the patients being nonverbal, some of the approaches are different, relying on caretaker questioning and/or animal behaviors and responses. Christiansen and Forkman [56] reviewed research articles utilizing QoL assessments in veterinary medicine, specifically in cats and dogs, and found that similarly to Gill and Feinstein [55], most of the assessments focused on health-related measures rather than taking a holistic approach to life quality. An example of a QoL assessment that includes a variety of parameters related to different aspects of animal well-being is the HHHHHMM assessment, scoring the following factors on a scale of 1 to 10: Hurt, hunger, hydration, hygiene, happiness, mobility, and more good days than bad [57]. In companion animal medicine, much of the discussion concerning QoL is focused on palliative care of pets to ensure that animal companions (ailing, sick, old) are provided with a satisfactory QoL, the justification for keeping them alive or electing to perform euthanasia. It has been emphasized that with the significant advancements in treatment options that can prolong life, it is necessary to have effective and meaningful ways of measuring QoL [1,58]; technological advancements have been able to substantially extend a pet’s life but at what cost to the quality of that animal’s life [59]?

Research and resources on practical and meaningful QoL assessments in cattle is lacking considerably, and arguably non-existent. There is significant discussion among stakeholders (veterinarians, animal scientists, and producers) about what animal welfare means to an animal, ensuring that either their Five Freedoms [60] are met or that we have made provisions for all of Fraser’s three orientations (basic health and functioning, affective states, and natural living) [61], but there is no suggestion of a consistent standardized tool to effectively assess the QoL of a dairy cow. Discussions about food animal welfare have certainly shifted from those of minimizing mistreatment to those of promoting positive experiences for animals [62], with care taken to provide animals with a life worth living. Within these discussions is a desire to outweigh the negative experiences with positive mental experiences and satisfaction of needs and expectations [63], but there is not a cohesive framework within which to do this regarding end-of-life decisions of dairy cattle. QoL assessments are not without limitations, but they still serve as helpful tools to inform veterinarians, caretakers, and producers about timely euthanasia decisions. This type of tool could prove helpful on dairies to provide a consistent measure of quality of life, but it would not be enough as there is one final puzzle piece to consider.

### 6.6. Human–Animal Bond

Traditionally when thinking about a human–animal bond, one thinks of pets who are often considered family members and does not as frequently consider the bond that is established between farm animals and their caretakers. Consequently, there exists considerably more research and discussion about human–animal relationships with companion animals as compared to food animals [64], although development in the area of understanding human–animal interactions in agriculture has been seen. The fact that food animals “are kept” because they produce something of value (e.g., milk and meat), rather than being kept because they are companions, cultivates the societal misconception that a bond between a livestock caretaker and an animal they care for either is weak or does not exist at all. In agriculture, research focuses more on human–animal interactions rather than on the human–animal bond itself. Studies aim to understand what factors, such as caretaker attitudes and personality traits, impact a caretaker’s interaction with the animals he/she works with and what the consequences of that interaction are on animal production, such as milk production and meat quality. Boivin et al. [65], Rushen and de Passille [66], Edwards-Callaway [67], and Hemsworth and Coleman [68] provide reviews of how stockmanship and human–animal interactions can positively and negatively impact both animal and worker well-being.

The AVMA considers a human-animal bond to be “a mutually beneficial and dynamic relationship between people and animals that is influenced by behaviors essential to the health and wellbeing of both. This includes, among other things, emotional, psychological, and physical interactions of people, animals, and the environment” [69]. The existence of a bond between dairy workers and the animals they care for is inevitable [70]. In personal communication with dairy workers, some have shared that there are certain dairy cows on the farm that greet them when they arrive for work. That is undeniably the sign of a bond; even if it was only a perception of the worker and not a reality, it is a significant driver in how the worker will approach decision-making regarding the cow’s welfare and place in the herd. Relationships between caretakers and the animals they care for certainly can have benefits for both parties, but in some instances, the caretaker’s attachment to an animal could also impact the person’s ability to make objective decisions regarding the needs of the animal [71], the timeliness of euthanasia decisions being an example. When a dairy cow is sick or in pain, the caretakers want to do everything they can to keep that animal alive—as keeping that animal alive is the overarching goal of their daily work and euthanasia may be viewed as a failure. In small animal medicine, it has been noted by veterinarians that one of the most stressful situations is managing a situation in which the “client wishes to continue treatment despite poor animal welfare” [72]. Although this is not a congruent example, it emphasizes the difficulty of those involved in the everyday care of animals, whether it be pets or dairy cattle, in sometimes making the best decision for the animal, simply because of their emotional investment. In some of the literature on quality of life assessment, the concept of a “proxy assessment” and its necessity in assessing quality of life in animals is discussed [59]. Often in companion animal medicine, the animal owners, or proxies, provide information about the pet’s experience and perceptions. This is arguably the same in food animals. Although the need for a proxy assessment is real in non-verbal animals, caretakers serving that role may often be biased in the opinion provided creating the need for a “proxy” on behalf of the cow independent of her daily caregiver. The genuine bond between a dairy worker and the animals he/she cares for, often taken for granted, may very well represent a significant contributor to the untimeliness of euthanasia seen on dairy farms.

## 7. What do We Recommend

Having identified six interconnected and complex factors that impede our success in ensuring timely euthanasia, the challenge to success is real but not insurmountable. What might the path to success look like? The simple answer to ensuring timely euthanasia is that every farm have written euthanasia guidelines that include who is responsible for performing it, how it will be carried out, descriptions of the appropriate methods, necessary equipment, and guidelines that state clearly when euthanasia should be considered as the best option to decrease animal suffering. However, as we have outlined above, there is nothing simple about managing timely euthanasia in any environment, whether it be on the farm or in the veterinary clinic. What is needed is a deliberate effort to shift the organizational culture of the veterinary profession and farm to ensure that timely euthanasia is viewed and practiced as a priority for the business and the animal in need of relief. Organizational culture evolves only if that evolution is supported by top management in terms of policies, resource allocation, and mechanisms for holding stakeholders accountable. For a cultural shift to occur around the management of timely euthanasia, it is essential that upper and middle management are engaged in the efforts listed below.

- Development and dissemination of training resources and clear guidelines on timely euthanasia.
  ○ In order to develop effective training resources and guidelines for timely euthanasia, stakeholders (caretakers, management, veterinarian) should be engaged and invited to provide input regarding content, delivery methods, language, and other logistics. Considering the audience and creating culturally congruent tools that are clear and accessible to all is imperative.
  ○ Written guidelines should provide enough information for caretakers to make medical treatment and euthanasia decisions. The final decision to euthanize an animal should not fall on the caretaker. Instead, guidelines should inform that decision in most cases. When exceptions occur, caretakers should be able to consult with management or the farm’s veterinarian. Caretakers should be aware of who they need to contact in case they need assistance with the decision-making process or euthanasia procedures.
  ○ Guidelines can aid in the development of clear decision trees that can assist in euthanasia decision-making. Decision trees allow for objective and consistent decision-making removing guesswork and bias from the process.
  ○ Training material should state clearly some conditions that necessitate immediate euthanasia. As stated, this has been done previously in the swine industry and this material could be used as a model for development in the dairy industry.
- Planned and regular training sessions and staff discussions.
  ○ The topic of euthanasia as a means to cease animal suffering and as a critical component of animal care should be first introduced during employee onboarding, regardless of position description. New employees with limited experience in animal husbandry or livestock production might not perceive euthanasia as a humane practice.
  ○ All farm employees directly or indirectly involved in animal care should be trained on (1) the importance of timely euthanasia (i.e., why?); (2) acceptable euthanasia methods and necessary equipment; (3) guidelines that clearly state when euthanasia should be considered as the best option to decrease animal suffering; (4) common diseases unlikely to respond to treatment, including but not limited to severe lameness, cancer eye, bloat, bloody gut, non-ambulatory cattle, chronic diarrhea, toxic mastitis intractable rectal/vaginal or uterine prolapse; (5) high-quality nursing care; (6) timely assessments to track acceptable treatment progress, disease progression, and changes in animal welfare considering quality of life; and (7) who is responsible for the euthanasia.
  ○ In addition to planned training interventions, management should have regular discussions with caretakers to clarify questions, address concerns, and discuss failures and successes.
  ○ Training interventions and staff meetings should stress the importance of caretaker wellbeing and mental health resources available for any employee who may need additional support.
  ○ Veterinarians should play an active and leading role in training and discussions about euthanasia with farm owners, managers, and caretakers.
- Measuring and tracking to help with objective assessment of procedures and accountability.
  ○ On-farm guidelines and procedures for timely euthanasia should be evaluated in order to continuously improve performance. Recording treatments, physical assessments, and other factors considered to euthanize an animal can help the farm’s management evaluate the timeliness of the process and the effectiveness of various management tools.
  ○ Euthanasia is a highly emotional process and biases based on the caretaker’s previous experiences or limited understanding can limit objectivity. Instead of relying on perceptions, developing a system to objectively measure the timeliness and effectiveness of our decisions will allow us to revise and improve the way animals are humanely treated.

## 8. Conclusions

Over one-half million dairy cattle unfit for transport are sent to slaughter annually and another one-half million calves suffer the process of dying. Failures of this magnitude represent a significant risk that extends far beyond the farm gate as public scrutiny and special interests seek to undermine consumer trust in agriculture and the dairy industry specifically. Despite its criticality, timeliness of euthanasia is not addressed specifically or defined in either the national dairy welfare program (FARM) or professional guidelines offered by the AABP. The lack of clarity and focus on the timeliness of euthanasia in guidance documents explains only part of the industry’s poor performance on this critical duty. It is essential that we understand and consider the daily pressures and conflicting priorities animal caretakers face, the lack of support and training, limited treatment options and challenges of evaluating treatment success and quality of life in addition to the emotional toll that euthanasia takes on every caretaker if we are going to create a successful path forward. It may be easy for some to disparage farms for making decisions in the best interest of profit (or break even) but doing so without considering the entire landscape, including tight margins and prevailing market disincentives, underestimates and oversimplifies the issue. If training, written protocols, and market disincentives were all that were needed to ensure timely euthanasia, surely the industry would have come closer than it is today. For the welfare of the individual cow and for the health and sustainability of the industry, it is paramount that stakeholders develop effective training and guidance tools that can be used at the farm level to ensure that farms support the creation of a culture of care that ensures that timely euthanasia is seen and practiced as a priority.

## Figures and Tables

**Table 1:** Estimates of the number of dairy cattle affected by, sold with, and died with severe conditions based on data reported in the 2014 National Animal Health Monitoring System (NAHMS) Health and Management Practices on U.S. Dairy Operations report [4]. Number of cows affected based on NAHMS estimates applied to the total number of lactating cows (9,399,000 head) in the US in 2018 [18].

Severe Conditions	% Cows Affected ^1^	Estimated # of Cows Affected ^2^	% Successful Recovery ^1^	% of Cows Removed (Not Euthanized) ^1^	# of Cows that Should Have Been Euthanized but Were Marketed ^2,3^
Cancer eye	0.20%	18,798	36.60%	36.20%	6805
Bloat	0.30%	28,197	54.50%	16.20%	4568
Bloody gut	0.30%	28,197	20.60%	36.10%	10,179
Downer	2.20%	206,778	22.40%	19.10%	39,495
Lame	16.80%	1,579,032	84.20%	14.70%	232,118
^4^ Severely lame	7.00%	657,930		5.90%	38,818
Total Affected					331,982

^1^ Values from [4]; ^2^ Calculated values using 9,399,000 head as total lactating dairy cow population; ^3^ These conditions are considered indicators of euthanasia in various industry guidelines, although provisions for timeliness in these guidelines is generally provided [5,8]; ^4^ Figures for severely lame cows are estimated based on published literature [17] as a percentage of reported lameness prevalence reported in the NAHMS data [4].

**Table 2:** Estimates of the number of dairy cattle by age group that die annually on dairy farms based on self-reported estimates of mortality and euthanasia rates reported in the 2014 NAHMS Health and Management Practices on U.S. Dairy Operations report [4]. Number of animals affected based on NAHMS estimates applied to the total number lactating cows, heifers and pre-weaned calves [18].

Age Group	% Mortality ^1^	Estimated # of Mortalities that Were Not Euthanized ^2^	% of Animals That Died without Assistance ^1^	Estimated # of Animals Euthanized on Farm ^2^	% of Animals Euthanized on Farm ^1^
Pre-weaned calves	6.40%	281,970	6%	18,798	0.40%
Heifer	1.90%	159,783	1.70%	18,798	0.20%
Lactating Cows	5.60%	300,768	3.20%	225,576	2.40%
Total		742,521		263,172	

^1^ Values from [4]; ^2^ Estimated values calculated using animal type populations data reported by USDA [18].
